# Superior Root Hair Formation Confers Root Efficiency in Some, But Not All, Rice Genotypes upon P Deficiency

**DOI:** 10.3389/fpls.2016.01935

**Published:** 2016-12-21

**Authors:** Josefine Nestler, Matthias Wissuwa

**Affiliations:** Crop, Livestock and Environment Division, Japan International Research Center for Agricultural SciencesTsukuba, Japan

**Keywords:** field, longevity, phosphorus, rice, root efficiency, root hair, root type

## Abstract

Root hairs are a low-cost way to extend root surface area (RSA), water and nutrient acquisition. This study investigated to what extend variation exists for root hair formation in rice in dependence of genotype, phosphorus (P) supply, growth medium, and root type. In general, genotypic variation was found for three root hair properties: root hair length, density, and longevity. In low P nutrient solution more than twofold genotypic difference was detected for root hair length while only onefold variation was found in low P soil. These differences were mostly due to the ability of some genotypes to increase root hair length in response to P deficiency. In addition, we were able to show that a higher proportion of root hairs remain viable even in mature, field-grown plants under low P conditions. All investigated root hair parameters exhibited high correlations across root types which were always higher in the low P conditions compared to the high P controls. Therefore we hypothesize that a low P response leads to a systemic signal in the entire root system. The genotype DJ123 consistently had the longest root hairs under low P conditions and we estimated that, across the field-grown root system, root hairs increased the total RSA by 31% in this genotype. This would explain why DJ123 is considered to be very root efficient in P uptake and suggests that DJ123 should be utilized as a donor in breeding for enhanced P uptake. Surprisingly, another root and P efficient genotype seemed not to rely on root hair growth upon P deficiency and therefore must contain different methods of low P adaptation. Genotypic ranking of root hair properties did change substantially with growth condition highlighting the need to phenotype plants in soil-based conditions or at least to validate results obtained in solution-based growth conditions.

## Introduction

Phosphorus (P) is an essential element for plant nutrition and deficiency in P supply can lead to growth reduction, developmental delays, and crop yield loss. Although soil P deficiency, which is present in 50% of agricultural soils (Lynch, 2011), can be reduced by fertilizer application this is not affordable for many resource-poor farmers in developing regions. Also, excess P can be washed out of soils eventually leading to water eutrophication (Raven and Taylor, 2003).

Aside of better P utilization efficiency within a plant, a cost-effective increase in crop yield under P limiting conditions can be achieved by improving P uptake from the soil (Ismail et al., 2007). This can be accomplished either through improved P mobilization in the soil or through increased soil exploration (reviewed in Richardson et al., 2009; Lynch, 2011; Rose et al., 2012). P mobility or availability can be enhanced by root exudation of low molecular-weight organic acids, protons, or phosphatases leading to higher P solubilization in the rhizosphere, or an increased production of P transporters. Production of finer roots, and longer or more root hairs lead to an increase of the root surface area (RSA) and therefore to improved soil exploration (Lynch, 2007).

Root hairs were directly (Gahoonia and Nielsen, 1998) and indirectly (Gahoonia et al., 1997) linked to P uptake from soil and model simulations suggested that root hair length is of greater importance for P uptake (Zygalakis et al., 2011) than root hair longevity and density (Brown et al., 2012). Although P deficiency can lead to increased root hair length and density in tomato (*Lycopersicon esculentum*), rape (*Brassica oleracea*), spinach (*Spinacia oleracea*) (Foehse and Jungk, 1983), common bean (*Phaseolus vulgaris*, Miguel et al., 2015), and *Arabidopsis thaliana* (Bates and Lynch, 1996) this is not a general observation for plant species or even among varieties of a species. Recently, it was demonstrated that P deficient soil can even lead to formation of shorter root hairs on rice (*Oryza sativa*) roots compared to sufficiently P supplied soil (Nestler et al., 2016). And among 166 Arabidopsis genotypes the majority showed no difference in root hair length in relation to P level (Stetter et al., 2015). In maize (*Zea mays*) on the other hand, several varieties have been shown to produce longer root hairs under low P conditions in the field, and in general long root hair genotypes were capable of better plant growth compared to plants with shorter root hairs (Zhu et al., 2010).

In contrast to Arabidopsis, where root hair initiation and outgrowth are rather well studied (reviewed in Hochholdinger and Nestler, 2013; Schiefelbein et al., 2014), few detailed investigations have been published for monocot crop species (recently reviewed in Marzec et al., 2015). In rice, several genes have been linked to root hair elongation including two *EXPANSIN* genes, *OsEXPA8* (Ma et al., 2013) and *OsEXPA17* (ZhiMing et al., 2011), the *CELLULOSE SYNTHASE-LIKE* gene *OsCSLD1* (Kim et al., 2007), *FORMIN HOMOLOGY1* (OsFH1, Huang et al., 2013a), a sec14-nodulin domain containing protein encoded by *OsSNDP1* (Huang et al., 2013b), the bHLH transcription factor *OsRHL1* (Ding et al., 2009), the auxin transporter *AUXIN1* (OsAUX1, Yu et al., 2015), and the transcription factor encoding *OsARF16* (Shen et al., 2013). In addition, the MYB transcription factor PHOSPHATE STARVATION RESPONSE (PHR) proteins have been linked to root hair elongation under P limiting conditions. The loss-of-function mutants of all three *OsPHR* genes produced shorter root hairs in Pi-free media while overexpression lines formed longer root hairs under sufficient P nutrition compared to wild-type plants (Guo et al., 2015).

For crops like rice a large number of traditional varieties exist and each may possess specific adaptations to the environmental stresses at their origin. As a result such traditional varieties may outperform modern varieties under certain stresses including nutrient deficiencies (Wissuwa and Ae, 2001; Tyagi et al., 2012) and could therefore serve as donors of traits and genes that would allow modern varieties to reach their yield potential at reduced fertilizer application (Wissuwa et al., 2016). Building on this idea, a recent study investigated root traits in a broad rice association panel under P deficient field conditions. Generally, performance under P deficiency was positively associated with root size, however, a small subset of accessions was identified as having high root efficiency (RE), meaning above-average P uptake with a rather small root system (Mori et al., 2016). The traditional variety DJ123 consistently ranked as having high RE, taking up 2.5-fold more P per cm^2^ of RSA compared to the modern variety IR64. One possible explanation for differences in RE could be genotypic differences in root hair formation. Recently it was found that root hair properties of rice genotypes differ depending on the growth media used. When grown in nutrient solution rice plants reacted to P deficiency by developing longer and denser root hairs, while a similar response to P supply was not detected in soil (Nestler et al., 2016). Furthermore, root hair development was primarily along main root axes in nutrient solution, whereas hair development on lateral roots was significant in soil-grown plants. In order to investigate underlying mechanisms leading to RE we examined root hair formation under P deficiency in various rice genotypes grown in different medium. The following points were addressed in this study: (i) are genotypic differences in root hair formation detectable under P deficient conditions; (ii) are these genotypic differences consistent across growth conditions and root types; (iii) can differences in root hair formation under P deficiency contribute to RE?

## Materials and Methods

### Plant Material and Preparation

Varieties belonging to the *aus* and *indica* sub-species of rice (*O. sativa* L.) and adapted to either lowland (IR64, Mudgo, Sadri Tor Misri, Taichung Native, Yodanya) or upland (DJ123, Kalubala Vee, Lemont, Nerica4, Santhi Sufaid) growth conditions were used for the experiments. Seed dormancy was broken by incubation at 50°C for 2 days. Seeds were surface-sterilized in 0.5% NaOCl 0.15 M KH_2_PO_4_ for 5 min followed by three times washing in distilled water for 10 min. Germination was initiated in distilled water at 30°C in the dark. For the upland field experiment unsterilized seeds, their dormancy broken, were directly sown and gently covered by soil.

### Nutrient Solution

Germinated seeds were transferred to mesh trays floating on deionized water 2 days-after-sowing (DAS). Iron (12 μM FeEDTA) and calcium (0.1 mM CaCl_2_) were added 5 DAS and the pH adjusted to 5.7. P treatments were started at 14 DAS when seedlings were transferred to 45 l containers filled with a third-strength Yoshida solution (Yoshida et al., 1972) containing P concentrations of either 1 μM (low P) or 100 μM (high P) NaH_2_PO_4_ 2H_2_O. At 21 DAS the solution was replaced by half-strength Yoshida solution and at 28 DAS by full-strength Yoshida solution and throughout the pH was adjusted to 5.7. The five replicates per genotype were grown in a completely randomized design per treatment. The experiment was performed in a greenhouse with temperature and relative humidity varying between 25–32°C and 30–50%, respectively. At 35 DAS all plants were harvested and the longest seminal root was stored in 50% ethanol at 4°C prior to microscopic root hair evaluation.

### Soil-Based Growth Conditions

We conducted three experiments in soil, using Rhizoboxes or upland and lowland field plots. Field sites are located in Tsukuba, Japan, and soil properties have been described elsewhere (Nestler et al., 2016). The same P deficient Andosol soil from the upland field plot was used to fill Rhizoboxes.

#### Rhizobox Experiment

For this experiment five genotypes were grown in Rhizoboxes, DJ123, Santhi Sufaid, Nerica4, Taichung Native, and Sadri Tor Misri. At 2 DAS the seedlings were transferred to Rhizoboxes (30 cm × 30 cm × 3 cm outer dimensions) containing the P deficient Andosol soil either supplied with N-P-K fertilizer (equivalent to 50-50-50 kg ha^-1^, high P) or with N-K fertilizer (equivalent to 50-0-50 kg ha^-1^, low P). Initially boxes were watered to field capacity; during the growth period plants were watered daily using an equal amount of water per box that gradually increased in order to keep soil moist but not flooded. Per genotype five replicates were grown in a completely randomized block design. The plants were grown under the same greenhouse conditions as described in the nutrient solution section. At 28 DAS root systems were extracted by gently washing off soil; shoot and root fresh weight were determined, and the longest main root was harvested and stored in 50% ethanol at 4°C prior to root hair analysis.

#### Upland Field Experiment

A high P and low P field plot was used and fertilized with N-P-K fertilizer at rates of 50-50-50 kg ha^-1^ (high P) or 50-0-50 kg ha^-1^ (low P). The same genotypes used for the Rhizobox experiment were grown in the upland field, with the only exception being Sadri Tor Misri, which was not available at the time of planting, and replaced by Mudgo, another lowland-adapted genotype with a similar root system and similar RE performance. Four rows of each genotype were sown in three randomized replicate blocks at each P treatment simultaneously with the 2014 field experiment published in Mori et al. (2016). For all measurements and both harvests (at 50 and 100 DAS) the middle two rows were utilized providing two border rows per genotype. At 50 DAS a 1 m deep trench was dug perpendicular to plant rows across the entire field site to allow access to the growing root systems. Intact roots were removed from the soil by washing and stored at 4°C in 50% ethanol prior to root hair evaluation. Shoots and roots were harvested, the latter comprising the root systems down to 20 cm soil depth. Dry weights were determined after oven-drying samples at 60°C for 4 days. At 100 DAS shoots (including any formed panicles) were harvested and roots were excavated from the top soil until 75 cm soil depth (three blocks of 25 cm × 20 cm × 10 cm) by soaking the soil blocks in water until roots could be removed, and root dry weight was determined as above. Root samples for root hair evaluation were obtained from the very top layer (5 cm deep), the entire top soil (up to 25 cm depth), medium (50 cm), and deep (75 cm) soil layers and either stored in 50% ethanol until root hair length and density analysis or used fresh for root hair longevity determination.

#### Lowland/Paddy Field Experiment

At 2 DAS seedlings were transferred to a greenhouse nursery bed before being transplanted to the paddy field at 22 DAS in three randomized replicate blocks. Only a field site with sufficient nutrition (supplied with 50-50-50 kg ha^-1^ N-P-K fertilizer) was available. Intact roots were gently removed from the flooded paddy field 28 days after transplanting (DAT), and stored at 4°C in 50% ethanol prior to root hair evaluation.

### Evaluation of Root Hair Formation and Longevity

For all experiments the longest seminal or crown root (main root) per plant was harvested and several 1 cm long sections were used for further analyses. These sections were in 1, 10, 15, 20, and 25 cm distance from the root tip, and per 1 cm section 4 images were taken for the main root and its subordinate lateral roots (the long L-type, the short S-type, and second order LRs if present). Root hair length and density determination were performed as described previously (Nestler et al., 2016).

Root hair longevity determination for 100 DAS upland field-grown samples was performed by immediately incubating root sections in 0.1x PBS pH 8.0 containing 40 μg ml^-1^ Neutral Red (Wako, Osaka, Japan), which is a widely used stain for cell viability (Ehara et al., 1996; Dubrovsky et al., 2006). After >1 h incubation the solution was replaced by 0.1x PBS pH 8.0 and the samples stored at 4°C for up to 7 days until microscopically evaluation of Neutral Red uptake in root hair cells, visual as red color within the root hair cells.

### Expression Analyses

For Rhizobox-grown plants the root zone 10–20 cm from the root tip of the second longest main root was harvested and immediately frozen in liquid Nitrogen. RNA was isolated using the RNeasy Plant Mini Kit (Qiagen, Hilden, Germany) following the manufacturers protocol. Up to 500 ng of isolated total RNA was reverse transcribed via the PrimeScript RT reagent Kit (Takara Bio Inc., Kusatsu, Japan), and quantitative RT-PCR performed on a CFX96 Real-Time PCR detection system (Bio-Rad^1^) using the SYBR Premix Ex Taq (Perfect Real Time) Kit (Takara Bio Inc., Kusatsu, Japan). Relative expression of target genes was determined as Δc(t) values compared to two reference genes, *OsEIF1a* (Os02g0300700) and *OsACT7* (Os05g0438800). Amplification efficiency for each oligonucleotide primer pair (sequences summarized in Supplementary Table S1) was determined by a dilution series employing a mixture of all cDNAs as matrix.

### Statistical Analyses

For all statistical analyses the Statistix 9 software (Analytical Software, Tallahassee, USA) was used. A general analysis of variance (ANOVA) was applied to compare treatment effects between P treatments, and among genotypes within each P treatment. Values obtained for root hair length and density in the different root sections could be averaged as the ANOVA P treatment × root section interaction term was not significant for all root types.

## Results

### Rice Genotypes Exhibit Variation in Root Hair Formation When Grown in Nutrient Solution

**FIGURE 1:**
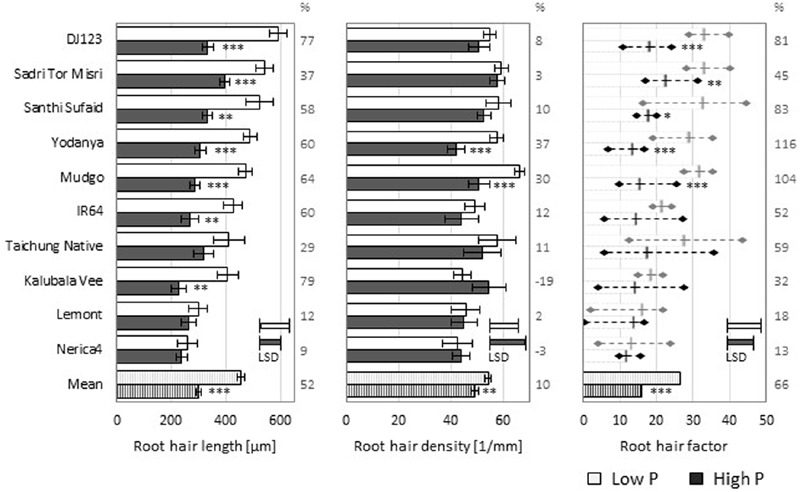
**Root hair properties of Nutrient solution-grown main roots.** Root hair length **(left)** and density **(middle)** means (*n* = 5) ± SE. The derived root hair factor (RHF, **right**), calculated as multiplication of root hair length and density, is shown as mean (*n* = 5) ± maximal/minimal replicate values as diamonds. Low P (light bars) and high P (dark bars). (%) values represent the increase in low P compared to high P. Statistical differences are shown per genotype as *P*-value ^∗^*P* ≤ 0.05, ^∗∗^*P* ≤ 0.01, ^∗∗∗^*P* ≤ 0.001 (P level); and LSD bars representing the least significant difference per P level (genotypic difference).

To investigate natural variation of rice root hair formation 10 genotypes varying in their RE under P deficiency (Mori et al., 2016) were grown in an easy to handle and reproducible nutrient solution setting. Average root hair length and density at low (1 μM) or high (100 μM) P supply are shown in **Figure 1** for main roots and in Supplementary Figure S1 for L-type LRs, S-type LRs, and second order LRs. Across genotypes P deficiency increased root hair length by 52% and root hair density by 10%. Estimating the total presence of root hairs by multiplying hair length and density yielded what we refer to as the root hair factor (RHF), which increased by 66% under P deficiency.

Growth in the low P nutrient solution increased root hair length for every genotype, while root hair density was increased for most, but decreased for two varieties compared to the high P condition. Nevertheless, the calculated RHF was increased in low P compared to high P nutrient solution for every tested genotype. The genotype DJ123 produced the longest root hairs in low P and it also showed the highest increase of root hair length compared to growth in high P nutrient solution (77%). Genotypic variation was lower for root hair density, with Mudgo and Sadri Tor Misri having the most root hairs when grown in the low P condition.

Root hair formation on the different lateral root classes (Supplementary Figure S1) varied from that on main roots (**Figure 1**). While the large L-type LRs formed considerable amounts of root hairs, the small LRs (S-type and second order LRs) produced very few or no root hairs at all. L-type and S-type LRs exhibited more and longer root hairs in the low P compared to the high P nutrient solution leading to higher values of RHF for both LR classes.

### Variations under Soil Conditions Is Decreased and Different Compared to Investigations in Nutrient Solution

To validate the results obtained in nutrient solution we chose a subset of genotypes previously found to contrast in RE in the field (Mori et al., 2016): Sadri Tor Misri, Mudgo (both with high P uptake, but low RE), and Taichung Native (less P uptake, low RE) as lowland-adapted types, and DJ123, Santhi Sufaid (both high P uptake, high RE), and Nerica4 (low P uptake, low RE) representing upland types.

**FIGURE 2:**
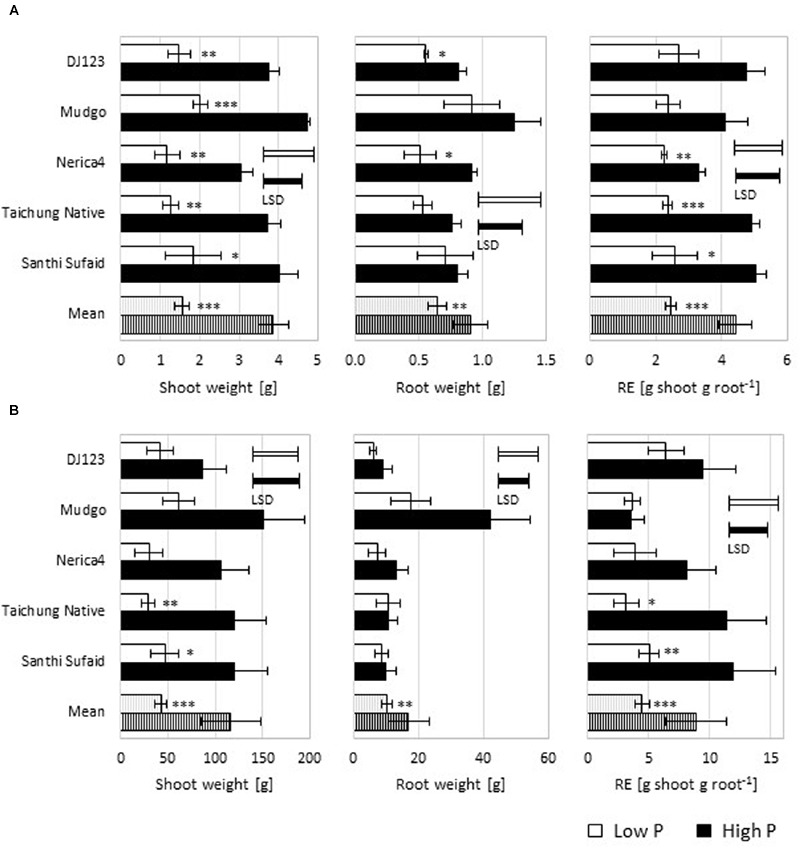
**Plant biomass and estimates of root efficiency (RE) in the upland field. (A)** Shoot and root biomass (dry weight; up to 20 cm field depth), and RE as shoot weight per root weight were measured 50 days-after-sowing (DAS) and are depicted as means (*n* = 3) ± SE. **(B)** Shoot dry weight, total root dry weight to a depth of 75 cm, and shoot weight per root weight were measured 100 DAS and are shown as means (*n* = 3) ± SE. Low P (light bars) and high P (dark bars). Statistical differences are shown per genotype as *P*-value ^∗^*P* ≤ 0.05, ^∗∗^*P* ≤ 0.01, ^∗∗∗^*P* ≤ 0.001 for P level differences; and the least significant difference (LSD) bars among genotypes within each P level.

Already at 50 DAS (**Figure 2A**) a large difference in biomass production was detectable between the high and low P upland field, with shoot and root dry weight lower in the latter. The ratio of shoot and root dry weights can be used as estimation of true RE (mg P uptake per cm^2^ RSA) as the respective correlations to P uptake and RSA are very high (Mori et al., 2016). Interestingly, while this simple estimate of RE (g shoot per g root) showed genotypic differences in the high P field, in the low P field no differences could be detected at this time point. Average rooting depth was decreased in the low P compared to the high P field and for both fields genotypic differences were detected (Supplementary Figure S2). Upland-adapted genotypes exhibited deeper roots in the high P field but under low P conditions this was not observed.

At 100 DAS (**Figure 2B**) the differences in shoot biomass production between the tested genotypes was similar to the 50 DAS time point. The produced root biomass up to 75 cm field depth was in a similar range for most genotypes, with the exception of Mudgo, producing almost four times the root biomass in high P compared to all other genotypes, which led to a very low RE. In the low P upland field DJ123 reached the highest RE followed by Santhi Sufaid, which is in line with the in-depth determination of RE performed earlier (Mori et al., 2016).

**FIGURE 3:**
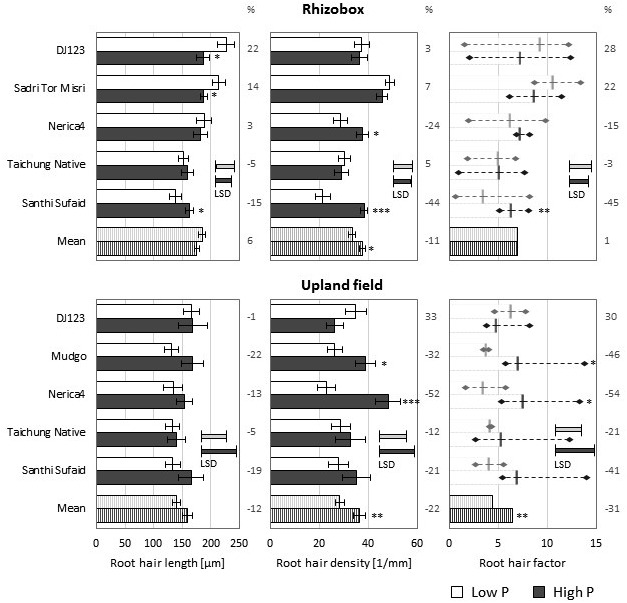
**Root hair properties on main roots of Rhizobox- and upland field-grown plants.** Root hair length **(left)** and density **(middle)** means (*n* = 5) ± SE are shown. The derived root hair factor (RHF, **right**) is visualized as mean (*n* = 5) ± highest/lowest replicate (diamond). Low P (light bars) and high P (dark bars). (%) represent the increase in low P compared to high *P*-values. Statistical differences are shown as *P*-value ^∗^*P* ≤ 0.05, ^∗∗^*P* ≤ 0.01, ^∗∗∗^*P* ≤ 0.001 for P level differences per genotype; and the LSD bars among genotypes within each P level.

While growth in both upland fields led to formation of comparably long root hairs on main roots their density was reduced in the low P field compared to the high P field (**Figure 3**). A similar decrease in root hair number was observed in Rhizoboxes, but in those root hair length was slightly increased in the low P condition. Over all genotypes the produced RHF was similar in the low and high P Rhizoboxes, however, when grown in the P deficient upland field the RHF was significantly lower compared to growth in the high P field. In Rhizoboxes only two genotypes, DJ123 and Sadri Tor Misri, did produce longer root hairs in P deficient soil, and these were also the only ones showing an increase in RHF values. In the upland field only DJ123 featured this increase in RHF, while all other genotypes exhibited decreased values compared to the high P field. The genotypic ranking compared to growth in nutrient solution was similar for the best performing genotypes: DJ123 forming the longest and Sadri Tor Misri the most dense root hairs in low P, however, other genotypes varied considerably between growth media. For instance, Nerica4, producing the shortest root hairs when grown in nutrient solution, did form medium long root hairs in low and high P soil (upland field and Rhizoboxes); while Santhi Sufaid, forming long root hairs in nutrient solution, exhibited the shortest root hairs in both low P soil conditions.

Although the total values were lower compared to those on the main roots, the lateral root types exhibited the same trends for genotypic differences as well as P level influence on root hair length and density in Rhizobox (Supplementary Figure S3) and upland field experiments (Supplementary Figure S4).

When root hair formation was investigated in lowland conditions (only a sufficiently P supplied field was available) a different genotypic ranking was found (Supplementary Figure S5). Nerica4, forming the shortest root hairs on nutrient solution-grown roots and medium long on soil-grown roots, did form the longest and most abundant root hairs in lowland conditions, leading also to the highest RHF. On the contrary, DJ123, with longest hairs in nutrient solution and upland soil, produced the shortest root hairs when grown in lowland conditions and also performed poorly regarding root hair density.

### Root Hair Vitality and Correlation of Root Hair Properties among Root Types

**FIGURE 4:**
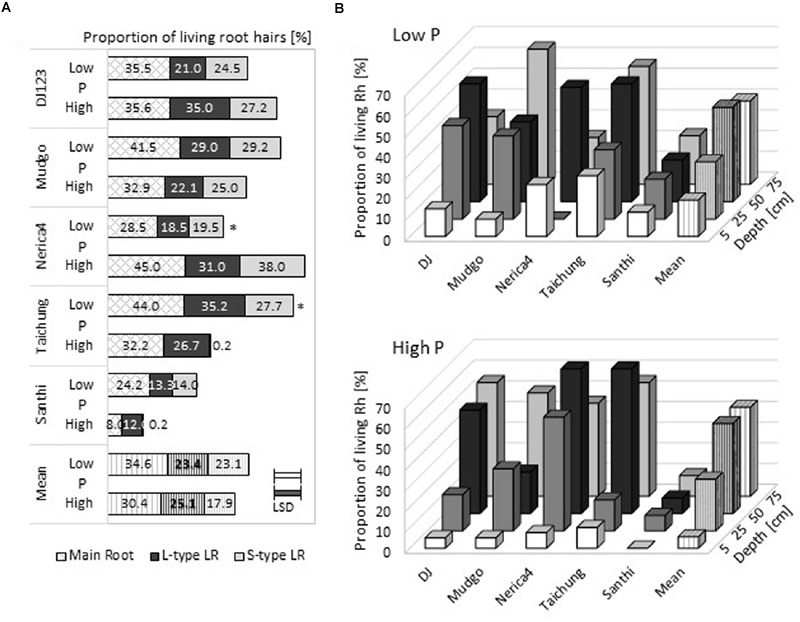
**Root hair liveliness in the upland field.** The relative amount of living root hairs in the upland field is depicted as means (*n* = 3). **(A)** The mean of all tested root depths for three different root types are shown. **(B)** Root hair liveliness distribution in the upland field on main roots in the low P field (upper graph), and the high P field (lower graph). Statistical differences are shown as *P*-value ^∗^*P* ≤ 0.05, for P level differences per genotype; and the LSD bars among genotypes within each P level.

In addition to root hair length and density, the viability of root hairs is of interest for nutrient uptake. Root samples from different field depths were harvested at 100 DAS in the upland field and analyzed on their capability of Neutral Red uptake, which is considered a vital stain (Ehara et al., 1996; Dubrovsky et al., 2006). On average over all genotypes and soil depths, a slightly higher proportion of root hairs was alive in the low P field at 100 DAS compared to the high P field (**Figure 4A**). Mudgo, Taichung Native, and Santhi Sufaid had higher proportions of viable root hairs in the P deficient field compared to the high P field while DJ123 and Nerica4 had more living root hairs in the high P upland field. The proportion of viable root hairs was similar among the different root types, but usually slightly higher for main roots. When root hair vitality was determined in relation to field depth, main roots in the very top soil (5 cm depth) exhibited the fewest living root hairs (**Figure 4B**). For both fields the proportion of viable root hairs increased with field depth, reaching similar, high values for 50 and 75 cm depth. This trend was observed for most genotypes and was also found for L-type and S-type LRs (Supplementary Figure S6). Santhi Sufaid consistently exhibited the lowest proportion of vital root hairs on all root types; on S-type LRs almost no living root hairs were found in the high P condition in any field depth.

Root hair parameters determined were analyzed for possible correlations in relation to genotype, P level, and root type. While root hair length correlated highly across different root types in the low P Rhizoboxes, no strong correlation between main and subordinate lateral roots was found in high P soil (Supplementary Table S2). Similar correlations were found, but with lower values, in the upland field (Supplementary Table S3). Root hair vitality was correlated on the different root types and was higher in the low P field compared to the high P field.

### Expression Analysis

**FIGURE 5:**
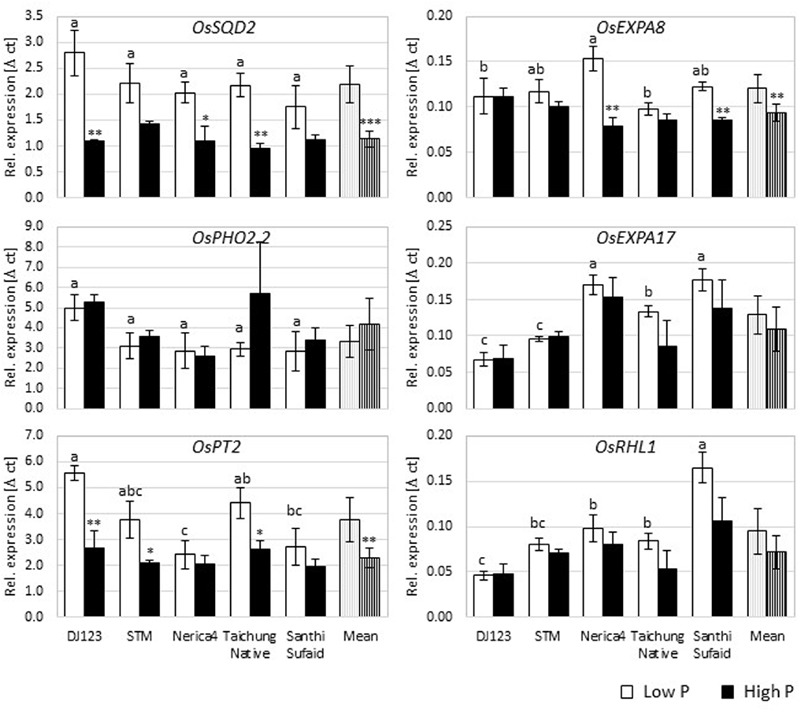
**Expression analyses in the Rhizobox experiment.** The relative expression [Δ c(t)] of selected P deficiency (*OsSQD2, OsPHO2.2, OsPT2*) or root hair (*OsEXPA8, OsEXPA17, OsRHL1*) related genes depicted relative to the reference genes *OsEIF* and *OsACT7*. Shown are mean values (*n* = 3) ± standard error. The student’s *t*-test was employed to calculate statistical differences between high and low P condition per genotype and gene (*P*-value: ^∗^*P* ≤ 0.05, ^∗∗^*P* ≤ 0.01, and ^∗∗∗^*P* ≤ 0.001), while the different letters indicate LSD among genotypes in the low P condition per gene.

Gene expression analyses for several rice genes known to be root hair- or P deficiency-related were performed in Rhizoboxes to gain insights into possible genotypic differences in the regulation of root hair formation in dependence of P (**Figure 5**; Supplementary Figure S7). These expression analyses were repeated with root samples obtained from the upland field yielding in similar results (data not shown). On average over all genotypes, *OsSQD2, OsPT1, OsPT2, OsPT6, OsPHR2, OsPHR3, OsEXPA8*, and *OsCSLD1* were upregulated under P deficiency, whereas expression of *OsPHO2, OsRHL1, OsEXPA17, OsPHR1, OsFH1, OsSNDP1, OsAUX1*, and *OsARF16* did not change significantly with P supply.

DJ123 as the genotype with longest root hairs and most pronounced increase in root hair length under P deficiency had the most pronounced change in gene expression for *OsSQD2* and *OsPT2*. While significant genotypic differences existed in expression of *OsPT2*, with DJ123 having higher expression compared to Nerica4 and Santhi Sufaid, differences in expression of *OsSQD2* were not significant. On the other hand, the expression level of *OsEXPA17* and *OsRHL1* under P limiting conditions was lowest in DJ123. Interestingly, the expression level of *OsAUX1* was similar across P levels and genotypes with the exception of DJ123 which produced much higher amounts of *OsAUX1* with sufficient P supply.

Furthermore, correlating the obtained expression levels with root hair length and density values in Rhizoboxes revealed strong correlation in low P, but low to none in high P (**Table 1**). For instance *OsPHR3* expression explained 60 and 84% of genotypic variation for root hair density and length under low P conditions, respectively, while only explaining 1 or 13% in high P. Expression of *OsARF16* and *OsPT2* was explaining the biggest proportion of variation for root hair density.

**Table 1:** Gene expression correlation (*r*^2^) with root hair length or density.

Gene	Area^∗^	*r*^2^ for root hair length		*r*^2^ for root hair density	
		Low P	High P	Low P	High P
*OsPHO2.1*	LP	**0.68**	0.00	0.26	0.19
*OsPT1*	LP	**0.64**	0.03	**0.77**	0.02
*OsSQD2*	LP	**0.63**	0.37	0.31	**0.93**
*OsPHO2.2*	LP	0.46	0.08	0.10	0.41
*OsPT2*	LP	0.25	0.00	0.20	0.39
*OsPT6*	LP	0.12	0.00	0.34	0.12
*OsPHR3*	LP, RH	**0.84**	0.13	**0.60**	0.01
*OsPHR2*	LP, RH	**0.50**	0.01	**0.60**	0.02
*OsPHR1*	LP, RH	0.08	0.05	0.40	0.11
*OsCSLD1*	RH	**0.70**	0.02	0.20	0.11
*OsRHL1*	RH	**0.65**	0.08	0.47	0.15
*OsEXPA17*	RH	**0.64**	0.02	0.20	0.11
*OsARF16*	RH	**0.61**	0.22	**0.80**	0.00
*OsSNDP1*	RH	0.26	0.00	0.33	0.18
*OsFH1*	RH	0.05	0.25	**0.50**	0.00
*OsEXPA8*	RH	0.01	0.33	0.05	0.10
*OsAUX1*	RH	0.00	0.14	0.18	0.09

*All data obtained from the Rhizobox experiment. ^∗^Area of known involvement per gene or its encoded protein. LP, response to low P; RH, root hair formation or elongation. The bolded values represent the r^*2*^ values which were significant.*

## Discussion

### Genotypic Variation of Root Hair Properties Is Altered in Different Growth Conditions and Can Attribute to Root Efficiency

When grown in nutrient solution most tested genotypes formed longer root hairs (**Figure 1**) and most tended to produce more root hairs in low P, both of which is generally perceived as a beneficial response to P deficiency (Lynch, 2007). Under low P conditions the variation in our experiments was much more enhanced ranging from short, 200 μm long root hairs on the main roots of Nerica4, to DJ123 with three times longer hairs. Much less genotypic plasticity could be found for root hair density in both P levels, which is in line with the modeling finding that root hair length is of greater importance for P deficiency response compared to root hair density (Zygalakis et al., 2011). Due to the small variations in root hair density the RHF values largely corresponded to root hair length. Little genotypic variation was detected under high P nutrition, possibly indicating that rice genotypes possess some basic level of root hair formation and that genotypic differences are more pronounced for the ability to increase root hair length under P deficiency. Still, cowpea (*Vigna unguiculata*) genotypes were shown to vary up to 65% in root hair length in soil without nutrient deficiency (Krasilnikoff et al., 2003).

Genotypes DJ123, Sadri Tor Misri, and Santhi Sufaid previously identified as having high P uptake (Mori et al., 2016) from the same low P field used here generated the highest RHFs in nutrient solution in our study. Soil-grown roots did not follow this trend completely (**Figure 3**) as Santhi Sufaid produced short and few root hairs in the upland field and even the shortest and fewest hairs in Rhizoboxes. Previously, we demonstrated the large impact of growth condition on root hair length and density leading to an overestimation of both properties on main roots in nutrient solution when compared to growth in soil (Nestler et al., 2016). Interestingly, this general observation varied between the tested genotypes. In the low P condition the best-performing genotypes in terms of root hair properties, DJ123 and Sadri Tor Misri, formed slightly less root hairs when grown in soil, but hair length was dramatically reduced to a third of what these genotypes reached in nutrient solution. In soil Nerica4 formed hairs of average length which almost reached the same extension as those grown in nutrient solution, thus indicating that Nerica4 might not have the potential to increase root hair length upon P deficiency. Nerica4 is a very popular variety in Africa but its performance under low soil fertility is poor (Koné et al., 2011). Our data highlights root hair length and density as potential target traits for improvement of P uptake in Nerica4 with varieties like DJ123 or Sadri Tor Misri being possible donors.

A detailed investigation of genotypic variation for RE in rice identified DJ123 and Santhi Sufaid as the most efficient genotypes in terms of P uptake per RSA under P deficiency (Mori et al., 2016). That study was performed simultaneously and at the same field site as our upland field experiment in 2014. At 100 DAS in the low P upland field we were able to confirm these results (**Figure 2B**), but interestingly at 50 DAS no difference could be detected between the tested genotypes (**Figure 2A**). Thus indicating that the underlying mechanisms eventually leading to RE in adult plants might be a rather small improvement in some mechanism that accumulates over the whole growth period as predicted through modeling (Wissuwa, 2005). Possible mechanisms underlying RE have been discussed by Mori et al. (2016) and our objective in this study was to test whether one of these potential mechanisms, the increase of RSA by longer and/or denser root hairs, could account for high RE in the studied genotypes. DJ123 was the most root-efficient genotype and the only one maintaining or increasing its root hair production in low P soil, suggesting that the resulting increase in RSA is indeed contributing to higher P uptake in this genotype. To illustrate the effect of root hairs on total RSA we roughly estimated the total root hair surface area (RHSA) by multiplying the RHF (root hair length × number per root type) times root hair circumference (assuming an average diameter of 20 μm). This was multiplied with the total length of each root type to estimate total RHSA, which was 37% larger in DJ123 (460 cm^2^) compared to Nerica4 (330 cm^2^) (Supplementary Figure S8). This RHSA increased the total RSA by 31 and 22%, respectively. Thus, given an identical root size, the production of slightly longer and denser hairs in field-grown DJ123 led to an almost 10% larger total RSA compared to Nerica4. The compound effect over time of having 10% higher RSA will depend on whether the additional P taken up will enable additional biomass accumulation and will therefore be considerably larger than 10% if P was the main growth-limiting factor (Wissuwa, 2005).

The situation is entirely different for Santhi Sufaid, the second root efficient genotype in this study. In both soil-based screening experiments it had a low and decreasing (relative to high P) RHF and the lowest proportion of living root hairs. Thus indicating that Santhi Sufaid is likely to rely on mechanisms enhancing P availability in the rhizosphere for its high RE. Therefore we can conclude that increased root hair formation is not a generally conserved mechanism in response to low P in soil. This is supported by observations from other plant species that also found inconsistent responses to root hair elongation in response to low P supply. Two common maize lines showed opposing reactions with line Mo17 extending root hairs twofold under P deficiency, whereas B73 had root hairs of equal length at high and low P supply (Zhu et al., 2005). Recently a large screen of Arabidopsis varieties under lab conditions also found that the majority of lines did not exhibit changed root hair production when facing P deficiency (Stetter et al., 2015).

### Gene Expression Correlates with Root Hair Formation in Low P

For gene expression analyses, several known P deficiency-response genes were chosen to confirm deficiency was detectable at the transcriptional level in our experiment, and to then investigate potential differences in P deficiency responses of the tested genotypes. As expected, the expression of most P-deficiency related genes such as *OsSQD2* (Oono et al., 2013) and *OsPT2* (Ai et al., 2009) was increased in low P compared to high P Rhizoboxes across genotypes, confirming P deficiency presence in this experiment on the molecular level (**Figure 5**; Supplementary Figure S7). Thus we can conclude that all tested genotypes responded in a similar, general fashion, and it can be emphasized that these genes are important for P uptake and transport not only under laboratory conditions, but also in P deficient soil. Some genotypic differences were detected, e.g., the expression pattern of several genes showed contrasting patterns in Nerica4 and DJ123. Although these genes were previously associated with P deficiency on a plant or root level, some even explained genotypic variation in root hair length or density (**Table 1**). This may indicate that, under low P conditions, the expression of P deficiency and/or starvation-response genes could lead to increased root hair formation, but further detailed experiments have to be conducted to verify this assumption.

On average over all genotypes, the expression of many root hair-associated genes was unrelated to P level which might be due to the sampling time or harvesting of whole root parts instead of root hairs. On the other hand, *OsEXPA8, OsPHR2, OsPHR3*, and *OsCSLD1* expression was increased in the low P Rhizoboxes for most genotypes suggesting their possible general involvement in P deficiency response in soil. Genotypic expression variation of *OsPHR3, OsPT1, OsARF16*, and *OsPHR2* explained 50–84% of genotypic root hair length and density variance specifically under low P conditions. Hence, these genes should be of particular interest in future investigations to further understand mechanisms leading to RE under P deficiency.

Interestingly, in low P soil the expression of the root hair-related genes *OsRHL1* and *OsEXPA17* was lower in the long hair-forming genotypes DJ123 and Sadri Tor Misri compared to the other genotypes. This might indicate a negative relationship of expression and root hair length formation in soil conditions or, more likely, may show residual expression of these two genes in roots, which covers or dilutes the root hair specific expression. To the best of our knowledge soil-grown root hairs have not been isolated for RNA extraction so far, but this would lead to interesting results regarding the expression level of these specialized cells in a natural environment.

While five genes reached high *r*^2^ values specifically for root hair length in low P, only a single one, *OsFH1*, explained 50% variation in root hair density without explaining root hair length variation. Root hairs of *Osfh1* mutants were demonstrated to be much shorter compared to wild-type root hairs in solution, and of similar length in agar (Huang et al., 2013a), but no difference in root hair density has been reported which, indicated by our results, might be of interest in response to P deficiency. Although all three transcription factors of the *OsPHR* family were linked to root hair formation under P deficiency or even starvation (Guo et al., 2015) in soil only two, *OsPHR2* and *OsPHR3*, exhibited increased expression in the low P condition compared to high P. *OsPHR1* might therefore not be involved in P deficiency response under natural conditions or its effect is not detectable at the root level at this plant age.

### The Proportion of Living Root Hairs Is Increased in Low P Soil

As younger root parts, closer to the root tip, are present in deeper soil layers in the field our finding of a higher proportion of living root hairs with increasing soil depths (**Figure 4**; Supplementary Figure S6) was expected. The biggest difference between low and high P fields was detected in the top soil layer (<5 cm depth), where root hairs in the high P field were almost entirely dead while 20% of hairs in the low P field were alive. This higher proportion of living root hairs under P deficiency might be due to following reasons: (i) these living root hairs are on newly formed young roots, (ii) field-grown root hairs live much longer than previously expected, or (iii) new root hairs were formed on older root sections. The first point will definitely contribute to some degree, but does not explain the difference in low and high P fields as in the latter more roots and more root hairs are produced and generally the plant lifecycle is longer. The second and third point seem to be more likely to be influenced by the local P level. Our data suggests that in the low P field environment root hairs live longer and thus are able to contribute longer to P uptake. Further investigations in this aspect of root and root hair property might show a previously unexpected plasticity of root hair formation, especially as, to the best of our knowledge, formation of root hairs on mature root parts far from the root tip has never been reported for rice.

### Correlated Root Hair Formation Indicates Genetic Programming

All of our root hair investigations, length, density, and living status, were examined on all available root types. The soil-based growth conditions, especially growth in Rhizoboxes and the upland field, yielded high similarities of root hair properties across all root types. Usually a gradient could be detected of shorter/less/lower proportion of living root hairs from main roots, through L-type and S-type to second order LRs, but the genotypic ranking remained similar or even unchanged. This was highlighted by the high correlation coefficients between root hair lengths on different root types (Supplementary Tables S2 and S3), specifically under low P conditions while little or no correlation was detected under high P conditions. Previously it was suggested that P supply in a split-pot system leads to a systemic, but also partly local, signal of P status (Rose et al., 2012). Although local low P concentrations induce local responses in terms of root hair production (Stetter et al., 2015) our data suggest that P deficiency led to a root system-wide response in root hair formation. In addition, as the tested genotypes varied in their correlation levels, we hypothesize that their genetic differences could mean: (i) the ability or characteristic for such a system response is different; (ii) the proportion of systemic and local response to P deficiency is different; and/or (iii) the plasticity of the whole root system might be different. All of these points will influence a genotypic P deficiency response and/or tolerance and will be part of RE and should be investigated in future experiments.

Growth in nutrient solution was the only conditions in which the root hair parameters varied greatly between the root types and genotypes, especially highlighted by near and complete absence of root hair formation on S-type and second order lateral roots, respectively. We detected this environmental impact previously (Nestler et al., 2016) and were able to confirm this being consistent across genotypes in the presented study. Screens for rice root hair properties performed in nutrient solution are therefore of limited value and have to be confirmed under more natural soil-based conditions.

## Conclusion

Genotypic variation was detectable for rice genotypes, but the growth condition influenced genotypic ranking and the range of variability. RE under low P conditions of the upland genotype DJ123 can be attributed in part to the formation of longer root hairs. Surprisingly, another root efficient upland genotype, Santhi Sufaid, showed continuous low performance in root hair properties thus it can be concluded that its RE might be related to other factors such as improved mycorrhiza colonization or exudation.

## Author Contributions

JN performed the research and analyzed the research data. JN and MW designed and interpreted the research work. JN and MW wrote the manuscript.

## Conflict of Interest Statement

The authors declare that the research was conducted in the absence of any commercial or financial relationships that could be construed as a potential conflict of interest.

## Footnotes

1 http://www.bio-rad.com

## References

[B1] Ai P. Sun S. Zhao J. Fan X. Xin W. Guo Q. (2009). Two rice phosphate transporters, OsPht1;2 and OsPht1;6, have different functions and kinetics properties in uptake and translocation. *Plant J.* 57 798–809. 10.1111/j.1365-313X.2008.03726.x 18980647

[B2] Bates T. R. Lynch J. P. (1996). Stimulation of root hair elongation in *Arabidopsis thaliana* by low phosphorus availability. *Plant Cell Environ.* 19 529–538. 10.1111/j.1365-3040.1996.tb00386.x

[B3] Brown L. K. George T. S. Thompson J. A. Wright G. Lyon J. Dupuy L. (2012). What are the implications of variation in root hair length on tolerance to phosphorus deficiency in combination with water stress in barley (Hordeum vulgare)? *Ann. Bot.* 110 319–328. 10.1093/aob/mcs085 22539540 PMC3394649

[B4] Ding W. Zhiming Y. Tong Y. Huang W. Chen H. Wu P. (2009). A transcription factor with a bHLH domain regulated root hair development in rice. *Cell Res.* 19 1309–1311. 10.1038/cr.2009.109 19752888

[B5] Dubrovsky J. G. Guttenberger M. Saralegui A. Napsucialy-Mendivil S. Voigt B. Baluška F. (2006). Neutral red as a probe for confocal laser scanning microscopy studies of plant roots. *Ann. Bot.* 97 1127–1138. 10.1093/aob/mcl045 16520341 PMC2803381

[B6] Ehara M. Noguchi T. Ueda K. (1996). Uptake of neutral red by vacuoles of a green alga, *Micrasterias pinnatifada*. *Plant Cell Physiol.* 37 734–741. 10.1093/pcp/pcn122

[B7] Foehse D. Jungk A. (1983). Influence of phosphate and nitrate supply on root hair formation of rape, spinach and tomato plants. *Plant Soil* 74 359–368. 10.1007/BF02181353

[B8] Gahoonia T. S. Care D. Nielsen N. E. (1997). Root hairs and phosphorus acquisition of wheat and barley cultivars. *Plant Soil* 191 181–188. 10.1023/A:1004270201418

[B9] Gahoonia T. S. Nielsen N. E. (1998). Direct evidence on participation of root hairs in phosphorus (32P) uptake from soil. *Plant Soil* 198 147–152. 10.1023/A:1004346412006

[B10] Guo M. Ruan W. Li C. Huang F. Zeng M. Liu Y. (2015). Integrative comparison of the role of the PHOSPHATE RESPONSE1 subfamily in phosphate signaling and homeostasis in rice. *Plant Physiol.* 168 1762–1776. 10.1104/pp.15.00736 26082401 PMC4528768

[B11] Hochholdinger F. Nestler J. (2013). “Genetics and genomics of plant root development,” in *Brenner’s Encyclopedia of Genetics* 2 Edn Vol. 5 eds Maloy S. Hughes K. (New York, NY: Elsevier Publishing) 349–352.

[B12] Huang J. Kim C. M. Xuan Y. Liu J. Kim T. H. Kim B. (2013a). Formin homology 1 (OsFH1) regulated root-hair elongation in rice (*Oryza sativa*). *Planta* 237 1227–1239. 10.1007/s00425-013-1838-8 23334469

[B13] Huang J. Kim C. M. Xuan Y. Park S. J. Piao H. L. Je B. I. (2013b). OsSNDP1, a Sec14-nodulin domain-containing protein, plays a critical role in root hair elongation in rice. *Plant Mol. Biol.* 82 39–50. 10.1007/s11103-013-0033-4 23456248

[B14] Ismail A. M. Heuer S. Thomson M. J. Wissuwa M. (2007). Genetic and genomic approaches to develop rice germplasm for problem soils. *Plant Mol. Biol.* 65 547–570. 10.1007/s11103-007-9215-2 17703278

[B15] Kim C. M. Park S. H. Je I. B. Park S. H. Park S. J. Piao H. L. (2007). OsCSLD1, a cellulose synthase-like D1 gene, is required for root hair morphogenesis in rice. *Plant Physiol.* 143 1220–1230. 10.1104/pp.106.091546 17259288 PMC1820921

[B16] Koné B. Amadji G. L. Aliou S. Diatta S. Akakpo C. (2011). Nutrient constraint and yield potential of rice on upland soil in the south of the Dahoumey gap of West Africa. *Arch. Agron. Soil Sci.* 57 763–774. 10.1080/03650340.2010.489554

[B17] Krasilnikoff G. Gahoonia T. Nielsen N. E. (2003). Variation in phosphorus uptake efficiency by genotypes of cowpea (*Vigna unguiculata*) due to differences in root and root hair length and induced rhizosphere processes. *Plant Soil* 251 83–91. 10.1023/A:1022934213879

[B18] Lynch J. P. (2007). Roots of the second green revolution. *Aust. J. Bot.* 55 493–512. 10.1071/BT06118

[B19] Lynch J. P. (2011). Root phenes for enhanced soil exploration and phosphorus acquisition: tools for future crops. *Plant Physiol.* 156 1041–1049. 10.1104/pp.111.175414 21610180 PMC3135935

[B20] Ma N. Wang Y. Qiu S. Kang Z. Che S. Wang G. (2013). Overexpression of OsEXPA8, a root-specific gene, improves rice growth and root system architecture by facilitating cell extension. *PLoS ONE* 8:e75997 10.1371/journal.pone.0075997 PMC3790854 24124527

[B21] Marzec M. Melzer M. Szarejko I. (2015). Root hair development in the grasses: what we already know and what we still need to know. *Plant Physiol.* 168 407–414. 10.1104/pp.15.00158 25873551 PMC4453783

[B22] Miguel M. A. Postma J. A. Lynch J. P. (2015). Phene synergism between root hair length and basal root growth angle for phosphorus acquisition. *Plant Physiol.* 167 1430–1439. 10.1104/pp.15.00145 25699587 PMC4378183

[B23] Mori A. Fukuda T. Vejchasarn P. Nestler J. Pariasca-Tanaka J. Wissuwa M. (2016). The role of root size versus root efficiency in phosphorus (P) acquisition of rice. *J. Exp. Bot.* 67 1179–1189. 10.1093/jxb/erv557 26842979

[B24] Nestler J. Keyes S. D. Wissuwa M. (2016). Root hair formation in rice (*Oryza sativa* L.) differs between root types and is altered in artificial growth conditions. *J. Exp. Bot.* 67 3699–3708. 10.1093/jxb/erw115 26976815

[B25] Oono Y. Kawahara Y. Yazawa T. Kanamori H. Kuramata M. Yamagata H. (2013). Diversity in the complexity of phosphate starvation transcriptomes among rice cultivars based on RNA-Seq profiles. *Plant Mol. Biol.* 83 523–537. 10.1007/s11103-013-0106-4 23857470 PMC3830200

[B26] Raven J. A. Taylor R. (2003). Macroalgal growth in nutrient enriched estuaries: a biogeochemical perspective. *Water Air Soil Pol.* 3 7–26. 10.1023/A:1022167722654

[B27] Richardson A. E. Hocking P. J. Simpson R. J. George T. S. (2009). Plant mechanisms to optimize access to soil phosphorus. *Crop Pasture Sci.* 60 124–143. 10.1071/CP07125

[B28] Rose T. J. Impa S. M. Rose M. T. Pariasca-Tanaka J. Mori A. Heuer S. (2012). Enhancing phosphorus and zinc acquisition efficiency in rice: a critical review of root traits and their potential utility in rice breeding. *Ann. Bot.* 112 331–345. 10.1093/aob/mcs217 23071218 PMC3698374

[B29] Schiefelbein J. Huang L. Zheng X. (2014). Regulation of epidermal cell fate in Arabidopsis roots: the importance of multiple feedback loops. *Front. Plant Sci.* 5:47 10.3389/fpls.2014.00047 PMC3925829 24596575

[B30] Shen C. Wang S. Zhang S. Xu Y. Qian Q. Qi Y. (2013). OsARF16, a transcription factor, is required for auxin and phosphate starvation response in rice (*Oryza sativa* L.). *Plant Cell Environ.* 36 607–620. 10.1111/pce.12001 22913536

[B31] Stetter M. G. Schmid K. Ludewig U. (2015). Uncovering genes and ploidy involved in the high diversity in root hair density, length and response to local scarce phosphate in *Arabidopsis thaliana*. *PLoS ONE* 10:3 10.1371/journal.pone.0120604 PMC4364354 25781967

[B32] Tyagi W. Rai M. Dohling A. (2012). Haplotype analysis for Pup1 locus in rice genotypes of north eastern and eastern India to identify suitable donors tolerant to low phosphorus. *SABRAO J. Breed. Genet.* 44 398–405.

[B33] Wissuwa M. (2005). Combining a modelling with a genetic approach in establishing associations between genetic and physiological effects in relation to phosphorus uptake. *Plant Soil* 269 57–68. 10.1007/s11104-004-2026-1

[B34] Wissuwa M. Ae N. (2001). Genotypic variation for tolerance to phosphorus deficiency in rice and the potential for its exploitation in rice improvement. *Plant Breed.* 120 43–48. 10.1046/j.1439-0523.2001.00561.x

[B35] Wissuwa M. Kretzschmar T. Rose T. J. (2016). From promise to application: root traits for enhanced nutrient capture in rice breeding. *J. Exp. Bot.* 67 3605–3615. 10.1093/jxb/erw061 27036129

[B36] Yoshida S. Forno D. A. Cock J. H. Gomez K. A. (1972). *Laboratory Manual for Physiological Studies of Rice* 2nd Edn. Los Baños: International Rice Research Institute 1–70.

[B37] Yu C. Sun C. Shen C. Wang S. Liu F. Liu Y. (2015). The auxin transporter, OsAUX1, is involved in primary root and root hair elongation and in Cd stress responses in rice (*Oryza sativa* L.). *Plant J.* 83 818–830. 10.1111/tpj.12929 26140668

[B38] ZhiMing Y. Bo K. XiaoWei H. ShaoLei L. YouHuang B. WoNa D. (2011). Root hair-specific expansins modulate root hair elongation in rice. *Plant J.* 66 725–734. 10.1111/j.1365-313X.2011.04533.x 21309868

[B39] Zhu J. Kaeppler S. M. Lynch J. P. (2005). Mapping of QTL controlling root hair length in maize (*Zea mays* L.) under phosphorus deficiency. *Plant Soil* 270 299–310. 10.1007/s11104-004-1697-y

[B40] Zhu J. Zhang C. Lynch J. P. (2010). The utility of phenotypic plasticity of root hair length for phosphorus acquisition. *Funct. Plant Biol.* 37 313–322. 10.1071/FP09197

[B41] Zygalakis K. C. Kirk G. J. D. Jones D. L. Wissuwa M. Roose T. (2011). A dual porosity model for nutrient uptake by root hairs. *New Phytol.* 192 676–688. 10.1111/j.1469-8137.2011.03840.x 21827499

