# Supplementary material for: Superior Root Hair Formation Confers Root Efficiency in Some, But Not All, Rice Genotypes upon P Deficiency
Source: Front Plant Sci. 2016 Dec 21;7:1935. doi: 10.3389/fpls.2016.01935 (PMC5174101; doi:10.3389/fpls.2016.01935)
Supplement: Supplementary file 1 [file Presentation_1.PDF]

Supplementary Material

Superior root hair formation confers root efficiency in some, but not all, rice genotypes upon P deficiency

Josefine Nestler[*], Matthias Wissuwa[*]

[*] Correspondence:

Matthias Wissuwa wissuwa@affrc.go.jp

Josefine Nestler j.nestler@fz-juelich.de

Included are:
Supplementary tables: 3
Supplementary figures: 8

Figure S1. Root hair properties on lateral roots of Nutrient solution-grown plants.
Root hair length (left) and density (middle) means (n=5) +/- SE. The derived root hair factor (RHF, right) is shown as mean (n=5) +/- maximal/minimal replicate value as diamond. Low P (light bars) and high P (dark bars). [%] values represent the increase in low P compared to high P. Statistical differences are shown per genotype with *P*-value *≤ 0.05, **≤ 0.01, ***≤ 0.001 for P level differences; and LSD bars representing the least significant difference among genotypes within each P level. Second order LRs are not depicted as no root hairs were found. n.d. = not determined.

Figure S2. Rooting depth in the upland field at 50 DAS.

Figure S3. Root hair properties on lateral roots of Rhizobox-grown plants.
Root hair length (left) and density (middle) means (n=5) +/- SE. The derived root hair factor (RHF, right) is shown as mean (n=5) +/- highest / lowest replicate (diamond). Low P (light bars) and high P (dark bars). [%] values represent the increase in low P compared to high P. Statistical differences are shown per genotype with *P*-value *≤ 0.05, **≤ 0.01, ***≤ 0.001 for P level differences; and LSD bars representing the least significant difference among genotypes within each P level.

Figure S4. Root hair properties on lateral roots of upland field-grown plants.
Root hair length (left) and density (middle) means (n=5) +/- SE. The derived root hair factor (RHF, right) is shown as mean (n=5) +/- maximal / minimal replicate value (diamond). Low P (light bars) and high P (dark bars). [%] values represent the increase in low P compared to high P. Statistical differences are shown per genotype with *P*-value *≤ 0.05, **≤ 0.01, ***≤ 0.001 for P level differences; and LSD bars representing the least significant difference among genotypes within each P level.

Figure S5. Root hair properties on all measured root types of lowland-grown plants.
Root hair length (left) and density (middle) means (n=5) +/- SE. The derived root hair factor (RHF, right) is shown as mean (n=5) +/- highest / lowest replicate (diamond). Low P (light bars) and high P (dark bars). [%] values represent the increase in low P compared to high P. Statistical differences are shown as least significant difference (LSD) bars among genotypes within high P. n.d. = not determined.

Figure S6. Root hair liveliness on lateral roots in the upland field.
Depicted are means (n=3) of the relative amount of living root hairs on lateral roots in increasing field depth.

Figure S7. Expression of selected genes in the Rhizobox experiment.
The relative expression [D c(t)] of selected P deficiency- (*OsPT1*, *OsPT6*, *OsPHO2.1*, *OsPHR1*, *OsPHR2*, *OsPHR3*) or root hair- (*OsAux1*, *OsARF1*, *OsCSLD1*, *OsFH1*, *OsSND1*) related genes is depicted relative to the expression of the reference genes *OsEIF1a* and *OsACT7*. Shown are mean values (n=3) +/- standard error. The student’s T-test was employed to calculate statistical differences between high and low P condition per genotype and gene (*P*-value: * ≤ 0.05, ** ≤ 0.01).

Figure S8. Total root hair surface area in upland soil under low P conditions.
The mean root hair surface area / cm root length was calculated by determining the surface area of a single hair per genotype and root type followed by multiplication of the specific root hair density. By integration of the average root type length composition in the upland field low P conditions (Nestler *et al*., 2016) the total root hair surface area was calculated for Nerica4 and DJ123.

Table S1. Oligonucleotide primer sequences.

Gene name	Accession number	Forward primer sequence	Reverse primer sequence	Product size [bp]
OsACT7	Os05g0438800	TCCAGCCATCTCTTGTTGGT	TCTTTCTGGTGGTGCAATCACT	221
OsARF16	Os06g09660	TAGACGAGAGCCCACGAGAA	TCGACATCCCTGTGATCCCT	199
OsAUX1	Os01g63770	ATCATGCACGCGATGTGGAA	CTGAGGCAGATGCTCTTGGT	293
OsCSLD1	Os10g0578200	GACGTCCCTCATGATACCGC	CAGACGTAGACGATGGTGGG	213
OsEIF1a	Os02g0300700	CAAGTACATGAACGACGAGGC	CCATTGGACTGATGAAGCAGC	193
OsEXPA8	Os01g0248900.1	CCTCCAAACTACGCCCTGT	GTCCCGGACCCCTTGATC	198
OsEXPA17	Os06g0108600	CACTACTGGTTGCTGCTCTAC	GTGCTGTAGGTGGTGAGCTTG	188
OsFH1	Os01g67240	AGATCACTGAGGTGTTGCGG	CGCAGAGTCACCATGGAAGT	185
OsPHO2.1	Os05g48390	AACTGCTGCTGTCCCTTCAA	TCTCGAAATGCTTTGGTGGC	186
OsPHO2.2	Os05g48390	CGGGTACATGGTTGTGTGGA	CCCTCGCAATCCGAAAGAGT	232
OsPHR1	Os03g21240	ACAGCAACTCCATCTGAGCC	TGCACTCTACGACGCTTCTC	203
OsPHR2	Os07g25710	TTGAGGAGCAAGGGAAGTGC	TGGATTCTCATGCCTGTTTGT	193
OsPHR3	Os02g04640	TGCCTCCATGAGTTAGTGCAG	TGTACTGGCAGAGATAGCACA	229
OsPT1	Os03g0150600.02	TCGTCATGTACGGATTCACC	ATCGTCCCGAGGAAGTTTGTG	160
OsPT2	Os03g0150800	TCGGTATGCTCATGACGCTG	ACCCCACAAATCCACAACTGTA	208
OsPT6	Os08g0564000	CAGGTGGTGGAGATGCGTAAT	CTGGAACAGGTTCTGGCTGT	162
OsRHL1	Os06g0184000	AGCGGAGCGGATGAAATCG	GAGAACCTTGACTTGGAGCTG	121
OsSNDP1	Os10g03400	GTTAGCCGCAACATGCCAAA	CCTCGATATCAGATAGGCGCT	154
OsSQD2	Os01g0142300	GCGATCTGGTACTGGAGGAA	TCTGTTGACCTCAGGTGCTG	93

Tables S2. Correlation analysis of Rhizobox-grown root hair properties.
Depicted are correlation coefficients of root hair length, density, root diameter, and root hair longevity proportions grown in Rhizoboxes. Low P (white) and High P (grey).

Correlation (R) between Root hair length on different root types

	MR	L-type LR	S-type LR	2nd Ord. LR
MR		0.2	0.04	0.15
L-type LR	0.88		0.73	0.57
S-type LR	0.65	0.8		0.65
2nd Ord. LR	0.62	0.64	0.68	

For n (Low P: 24, High P: 23) the *P*-value of correlation is ≤ 0.05 at R > 0.41.

Correlation (R) of root hair length with Rh number & root diameter on different root types

	MR	L-type LR	S-type LR	2nd Ord. LR
Rh number	0.83	0.62	0.39	0.28
Rh number	0.8	0.58	0.52	0.35
Rt diameter	0.82	0.69	0.66	0.46
Rt diameter	0.58	0.27	0.41	0.36

For n (Low P: 24, High P: 23) the *P*-value of correlation is ≤ 0.05 at R > 0.41.

Correlation (R) of root hair length with Rh number & root diameter per genotype

	DJ123	STM	Nerica4	Taichung	Santhi
Rh number	0.83	0.25	0.76	0.51	0.51
Rh number	0.41	-0.18	0.26	0.25	0.06
Rt diameter	0.73	0.88	0.47	0.82	0.54
Rt diameter	0.88	0.86	0.08	0.75	0.86

For n (Low P: 19, High P: 18) the *P*-value of correlation is ≤ 0.05 at R > 0.47.

Table S2

Tables S3. Correlation analysis of upland field-grown root hair properties.
Depicted are correlation coefficients of root hair length, density, root diameter, and root hair longevity proportions grown in the upland field for 100 DAS. Low P (white) and High P (grey).

Correlation (R) between Root hair length on different root types

	MR	L-type LR	S-type LR	2nd Ord. LR
MR		0.26	0.58	0.26
L-type LR	0.59		0.33	0.39
S-type LR	0.63	0.59		0.36
2nd Ord. LR	-0.16	0.30	0.33	

For n (Low P: 15, High P: 19) the *P*-value of correlation is ≤ 0.05 at R > 0.5.

Correlation (R) between Root hair liveliness on different root types

	MR	L-type LR	S-type LR	2nd Ord. LR
MR		0.76	0.74	n.d.
L-type LR	0.87		0.69	n.d.
S-type LR	0.93	0.90		n.d.
2nd Ord. LR	n.d.	n.d.	n.d.	

For n (Low P: 29, High P: 24) the *P*-value of correlation is ≤ 0.05 at R > 0.38.

Correlation (R) of root hair length with Rh number & liveliness on different root types

	MR	L-type LR	S-type LR	2nd Ord. LR
Rh number	0.74	0.83	0.82	0.84
Rh number	-0.8	0.40	0.27	0.67
Rh liveliness	-0.55	-0.24	-0.43	n.d.
Rh liveliness	-0.54	-0.72	-0.49	n.d.

For n (Low P: 29, High P: 24) the *P*-value of correlation is ≤ 0.05 at R > 0.42.

Correlation (R) of root hair length with Rh number & liveliness per genotype

	DJ123	Mudgo	Nerica4	Taichung	Santhi
Rh number	0.68	0.89	0.81	0.66	0.73
Rh number	0.81	0.73	0.47	0.84	0.83
Rh liveliness	0.29	-0.26	-0.08	-0.26	-0.06
Rh liveliness	-0.54	-0.45	-0.42	-0.05	0.60

For n (Low P: 18, High P: 21) the *P*-value of correlation is ≤ 0.05 at R > 0.46.

Table S3

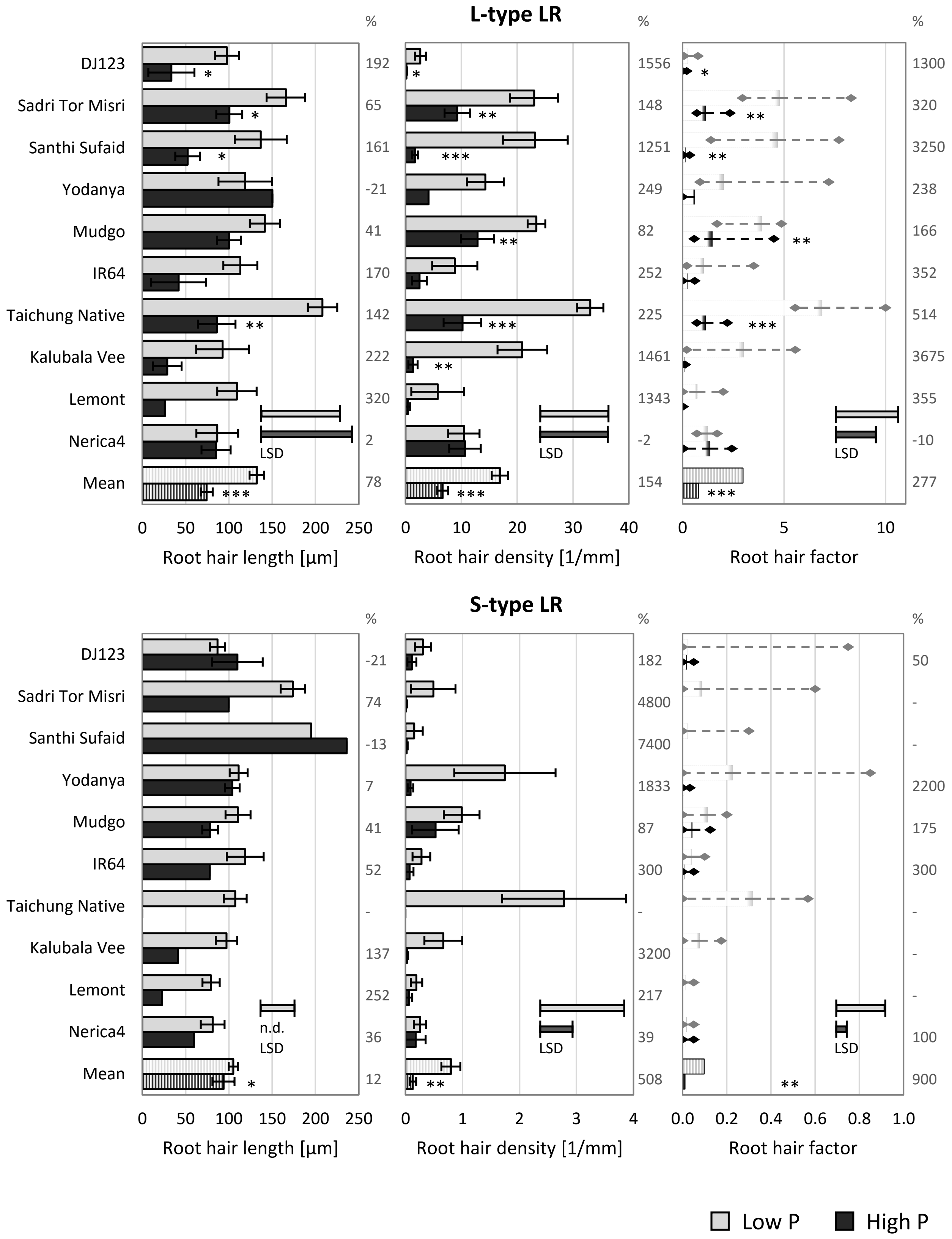

L-type LR
DJ123
Sadri Tor Misri
Santhi Sufaid
Yodanya
Mudgo
IR64
Taichung Native
Kalubala Vee
Lemont
Nerica4
Mean
%
192
65
161
-21
41
170
142
222
320
2
78
1556
148
1251
249
82
252
225
1461
1343
-2
154
1300
320
3250
238
166
352
514
3675
355
-10
277
LSD
Root hair length [µm]
Root hair density [1/mm]
Root hair factor
S-type LR
-21
74
-13
7
41
52
-
137
252
36
12
182
4800
7400
1833
87
300
-
3200
217
39
508
50
-
-
2200
175
300
-
-
-
100
900
n.d.
Low P
High P

Figure S1

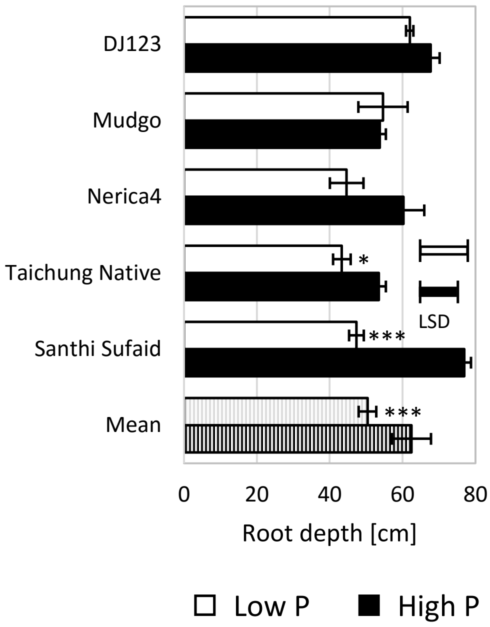
DJ123
Mudgo
Nerica4
Taichung Native
Santhi Sufaid
Mean
*
***
***
LSD
0
20
40
60
80
Root depth [cm]
Low P
High P

Figure S2

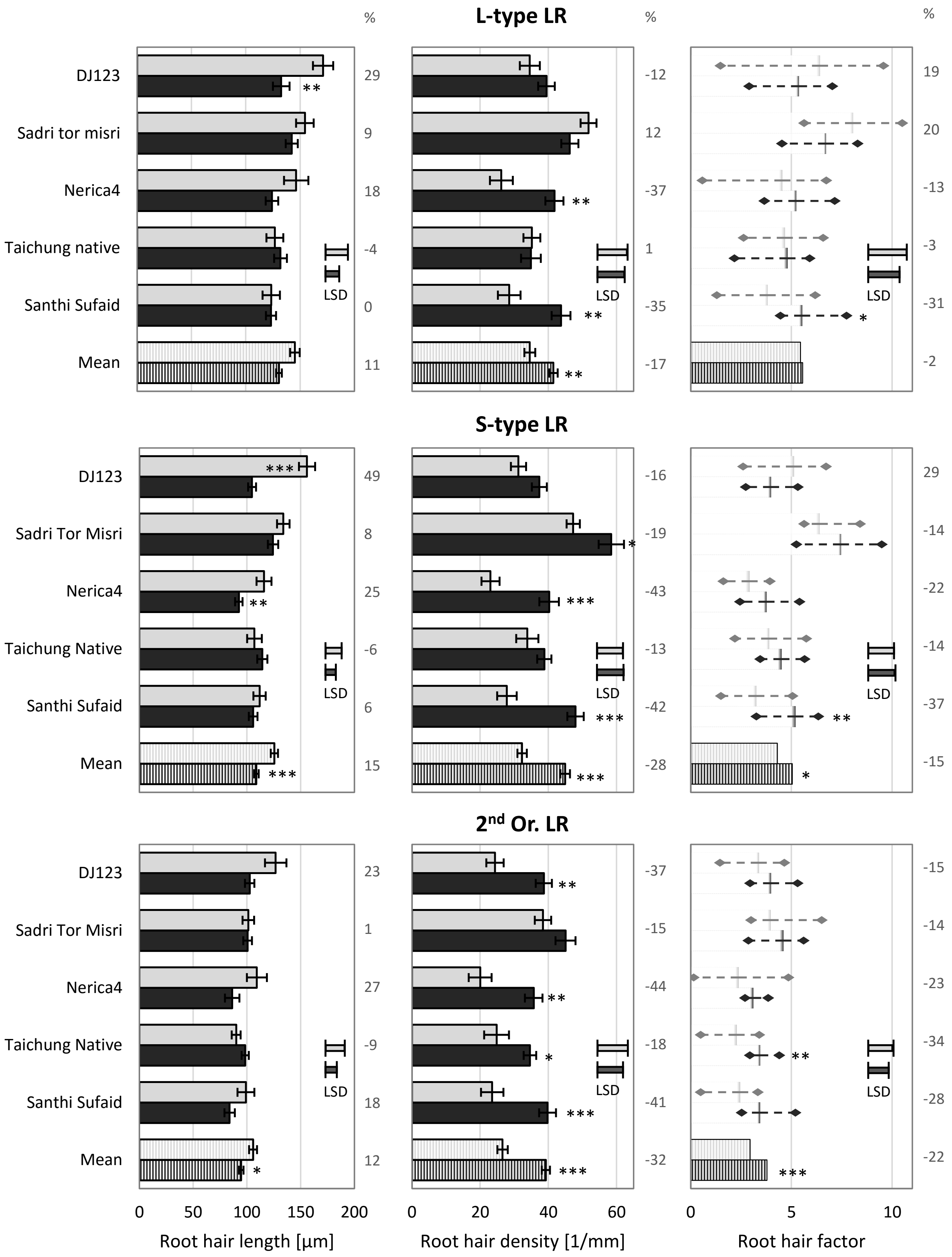
L-type LR
S-type LR
2nd Or. LR
%
DJ123
Sadri tor misri
Nerica4
Taichung native
Santhi Sufaid
Mean
Sadri Tor Misri
Taichung Native
29
9
18
-4
0
11
-12
12
-37
1
-35
-17
19
20
-13
-3
-31
-2
49
8
25
-6
6
15
-16
-19
-43
-13
-42
-28
29
-14
-22
-14
-37
-15
23
1
27
-9
18
12
-37
-15
-44
-18
-41
-32
-15
-14
-23
-34
-28
-22
LSD
0
50
100
150
200
20
40
60
5
10
Root hair length [µm]
Root hair density [1/mm]
Root hair factor
Low P
High P

Figure S3

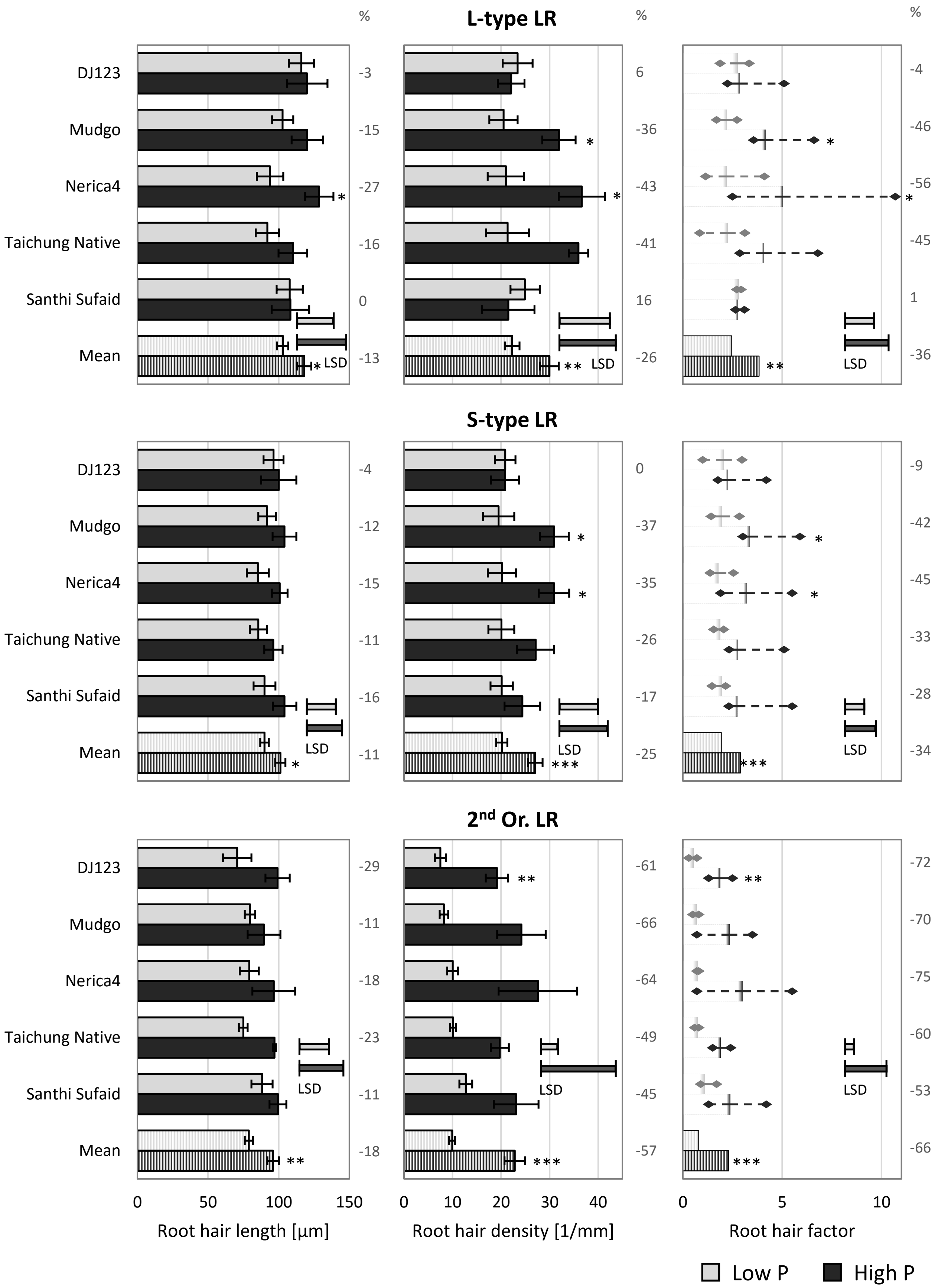
L-type LR
S-type LR
2nd Or. LR
%
DJ123
Mudgo
Nerica4
Taichung Native
Santhi Sufaid
Mean
LSD
Root hair length [µm]
Root hair density [1/mm]
Root hair factor
-3
-15
-27
-16
0
-13
6
-36
-43
-41
16
-26
-4
-46
-56
-45
1
-36
-4
-12
-15
-11
-16
-11
0
-37
-35
-26
-17
-25
-9
-42
-45
-33
-28
-34
-29
-11
-18
-23
-11
-18
-61
-66
-64
-49
-45
-57
-72
-70
-75
-60
-53
-66
Low P
High P

Figure S4

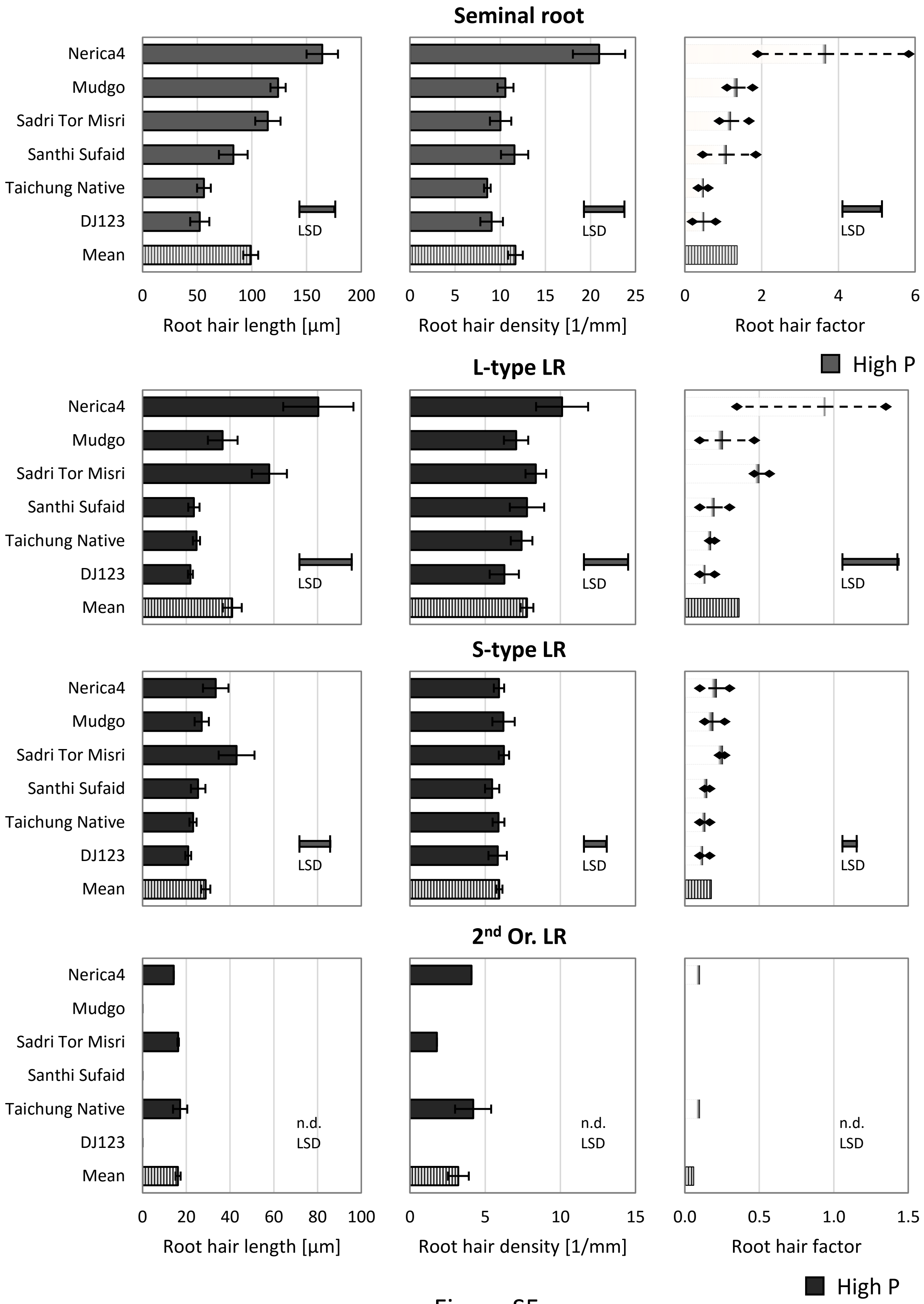
Seminal root
L-type LR
S-type LR
2nd Or. LR
Nerica4
Mudgo
Sadri Tor Misri
Santhi Sufaid
Taichung Native
DJ123
Mean
LSD
n.d.
Root hair length [µm]
Root hair density [1/mm]
Root hair factor
High P
0
50
100
150
200
5
10
15
20
25
2
4
6
40
60
80
0.0
0.5
1.0
1.5

Figure S5

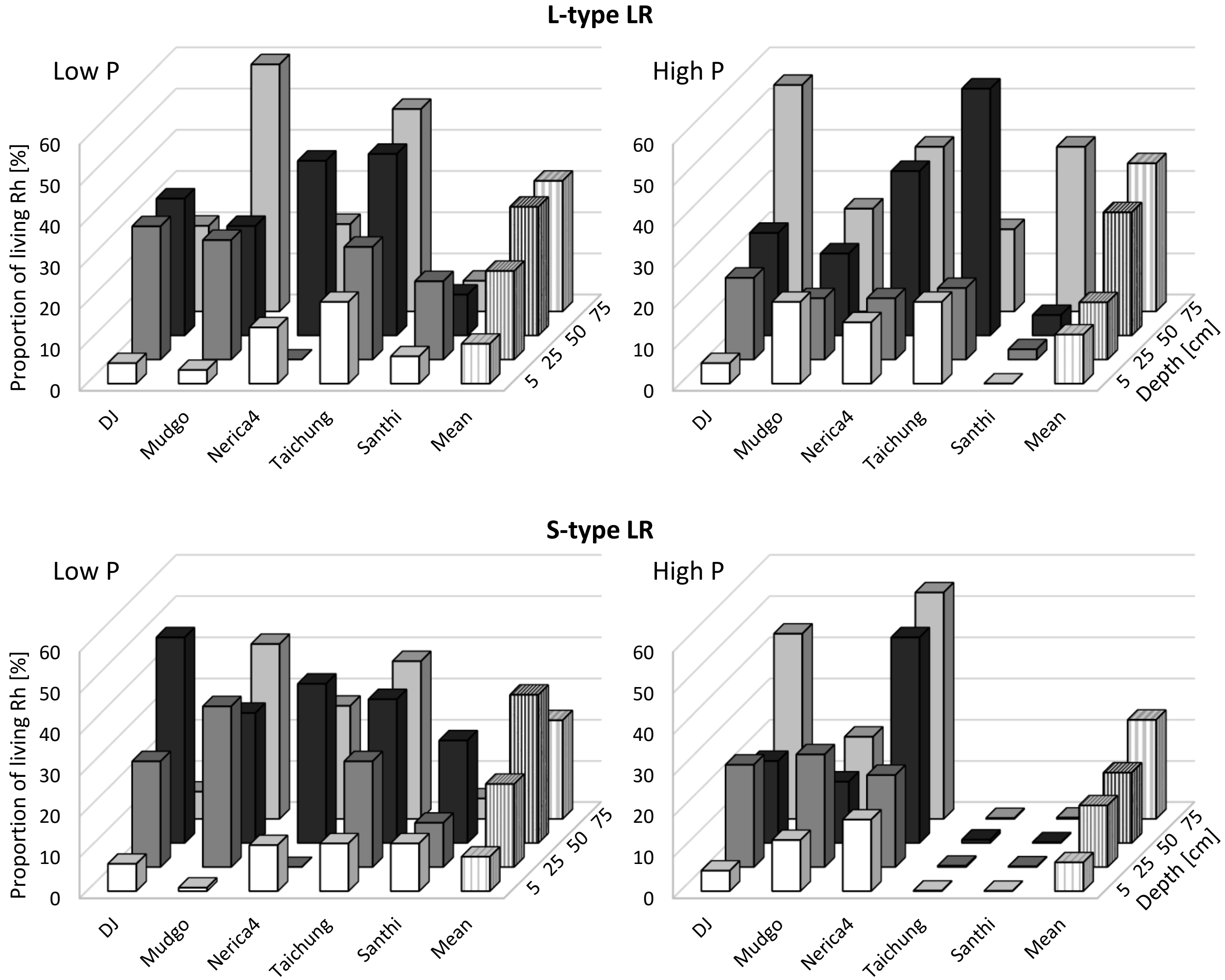

L-type LR
Low P
High P
S-type LR
Low P
High P
Proportion of living Rh [%]
0
10
20
30
40
50
60
DJ
Mudgo
Nerica4
Taichung
Santhi
Mean
5 25 50 75
Depth [cm]

Figure S6

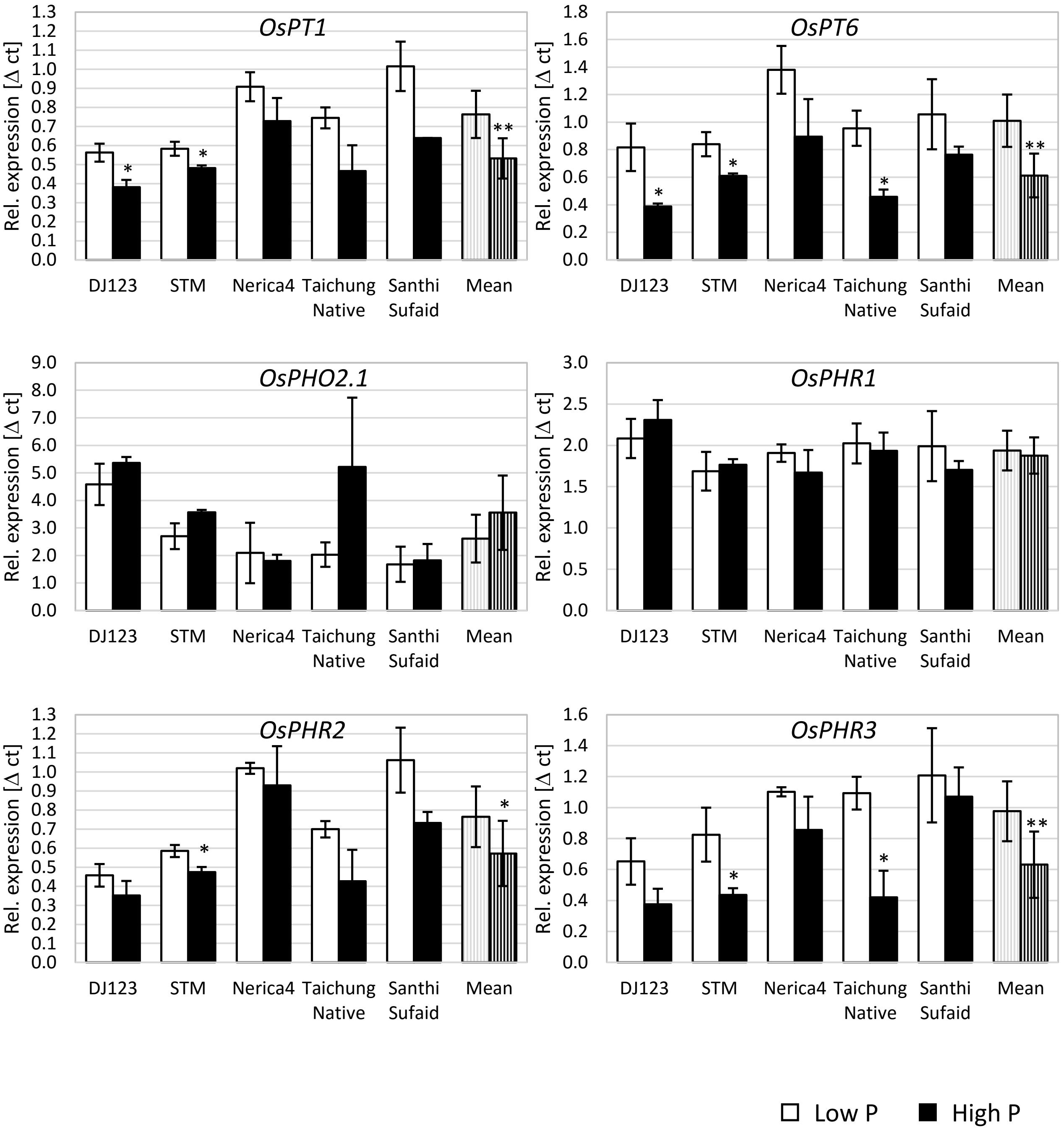
OsPT1
OsPT6
OsPHO2.1
OsPHR1
OsPHR2
OsPHR3
Rel. expression [Δ ct]
DJ123
STM
Nerica4
Taichung Native
Santhi Sufaid
Mean
Low P
High P

Figure S7

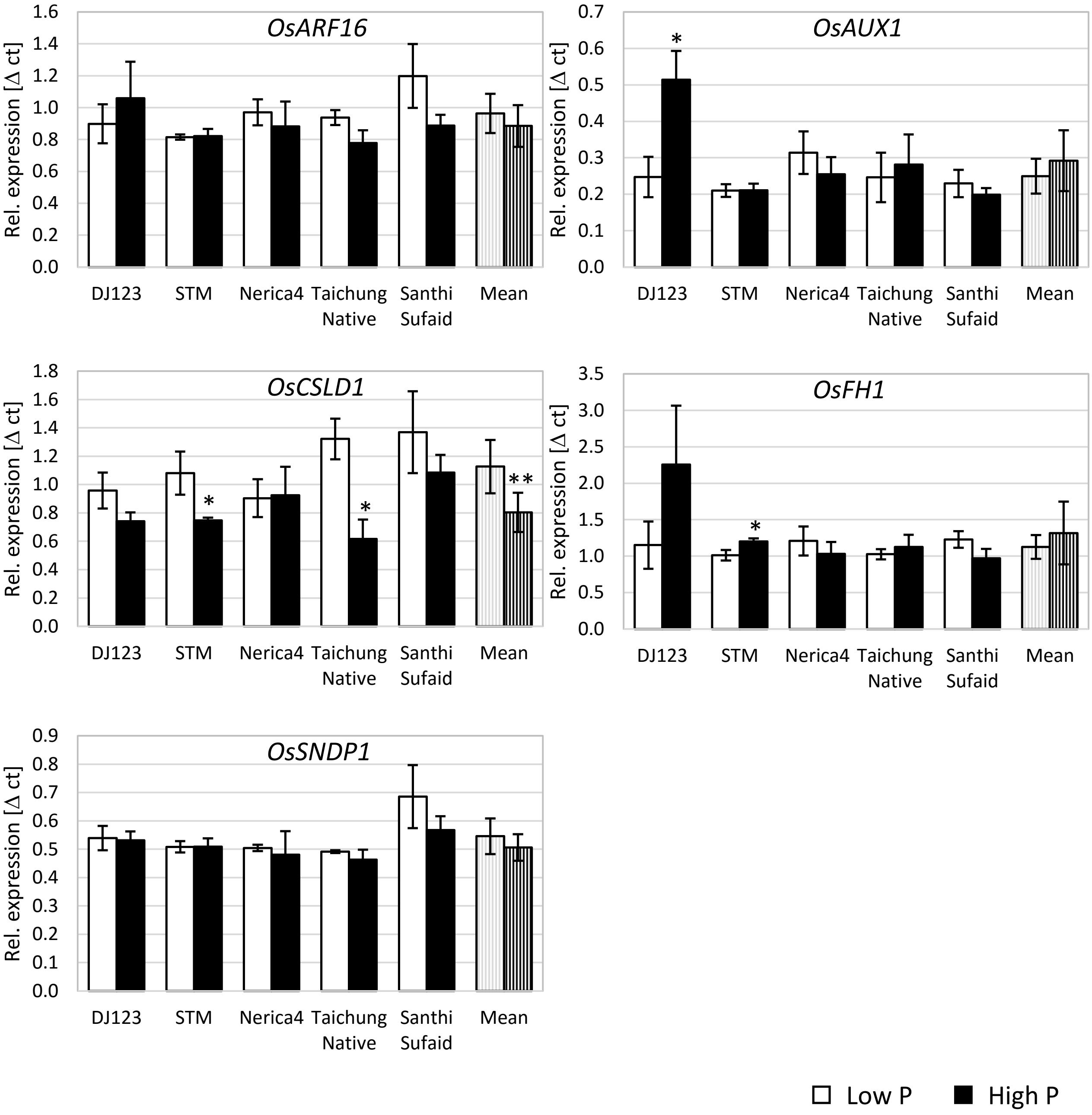

Figure S7 cont.

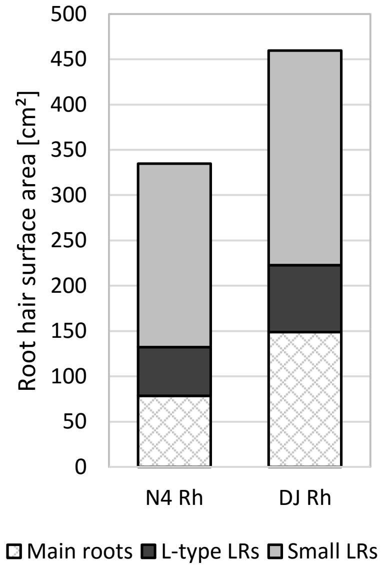

500
450
400
350
300
250
200
150
100
50
0
Root hair surface area [cm²]
N4 Rh
DJ Rh
Main roots
L-type LRs
Small LRs

Figure S8
